# Environmental DNA Reveals the Fish Community Structure Exhibited Instability and Trend of Miniaturization in the Xijiang River Basin of the Guizhou

**DOI:** 10.1002/ece3.71825

**Published:** 2025-09-19

**Authors:** Xiuhui Ma, Fujiang Huang, Ruiyuan Zhang, Tianhong Liu, Tianyang Zhang, Peng Zeng

**Affiliations:** ^1^ School of Animal Science Guizhou University Guiyang Guizhou China; ^2^ Key Laboratory of Animal Genetics, Breeding and Reproduction in the Plateau Mountainous Region, Ministry of Education Guizhou University Guiyang China; ^3^ Fisheries Research Institute, Academy of Agricultural Sciences of Guizhou Guiyang Guizhou China

**Keywords:** diversity, environmental DNA, environmental factors, fish community, Xijiang river

## Abstract

The seasonal monitoring of fish communities was conducted in the Xijiang River Basin of Guizhou Province using environmental DNA (eDNA) metabarcoding technology. A total of 97 fish species were identified during the 2023 survey, representing a detection rate of 61.94% compared to historical records. The findings demonstrated the following: (1) Fish communities exhibited significant spatiotemporal heterogeneity, with species diversity and composition varying seasonally and spatially; (2) human activities, such as reservoir construction, have led to the potential trend toward miniaturization of fish populations and pose a severe threat to the survival of indigenous species; (3) water *NH*_*3*_ *‐N*, *altitude,* and *pH* emerged as the key environmental factors influencing community structure. This research elucidated the dynamic characteristics of riverine fish communities in karst mountainous regions and provided a scientific foundation for the adaptive management of river basin ecosystems. Furthermore, this study represents the validation of eDNA technology's monitoring efficiency in karst areas characterized by complex geographical environments, thereby confirming its application value in assessing biodiversity in mountainous aquatic systems.

## Graphical abstract

Monitoring of fish communities is of great significance for the management and sustainability of river ecosystems. Environmental DNA (eDNA) metabarcoding technology has been demonstrated to be a highly sensitive biomonitoring tool. Nevertheless, it has seldom been tested in mountainous rivers within the geographically complex Karst region. We employed environmental DNA (eDNA) technology to carry out seasonal fish surveys in the Xijiang River basin of Guizhou province in 2023. The aim of this study was to investigate the characteristics of the fish community structure and diversity pattern. It provides a scientific basis for future protection and management.

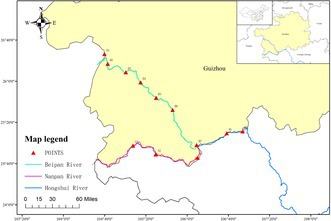

## 1 Introduction

Due to the impact of long‐term human activities, the deterioration of freshwater aquatic habitats and the reduction of aquatic living resources are conspicuous (Jia et al. 2024). Fish diversity constitutes an essential part of river ecosystems (Su et al. 2021). Fish are also a significant indicator of the aquatic environment, covering a spectrum of nutrient levels from zooplankton and herbivores to top predators, and are mobile while being highly sensitive to human disturbance (Karr et al. 1986; Matthews 1998; Stat et al. 2019). Hence, the distribution and abundance of fish are of vital importance for evaluating river ecosystems and implementing effective fisheries resource management (Rullens et al. 2021).

The structure and diversity of fish communities exhibit a highly intricate and intertwined relationship with the ecological environment of the aqueous habitats they occupy (Heino et al. 2015). Mountain rivers, serving as essential connectors that establish a nexus between highland and lowland ecosystems, play a pivotal role in the translocation and sequestration of nutrients (Wohl 2000). These rivers are characterized by salient and remarkable features, namely, steep topographical gradients, torrential water fluxes, copious hydro‐energy endowments, and a dynamic natural landscape. Such distinctive attributes render them not only prime for hydropower exploitation initiatives but also conducive habitats wherein a plenitude of scarce and indigenous fish species can grow and reproduce (Qiang 2012). Over the extended temporal span of natural selection pressures and historical evolutionary trajectories, fish have evolved sophisticated morphological architectures and elaborate life‐history phenotypes that are exquisitely attuned and adapted to the idiosyncratic and specific ecological context of mountain rivers. Consequently, any modifications to their habitats instigated by the establishment of cascade hydropower projects exert a significant impact on the fish populations resident within mountain rivers (Barbarossa et al. 2020). The erection of dams invariably precipitates elevated water levels within reservoirs, concomitant with decelerated flow velocities and a disrupted continuity of the riverine system. This, in turn, imposes severe spatial constraints on the inhabitable domain available for fish, culminating in a discernible reduction in fish abundances, a diminished genetic heterogeneity, and an emergent and concerning trend towards a homogenized composition of the fish community (Pringle et al. 2000).

The traditional approach involves accurately documenting species, quantities, and locations based on standardized field investigations to generate scientific outcomes (Jiang et al. 2024). However, the traditional method presents evident limitations in practical applications. Conventional fish survey techniques, such as gillnetting, electrofishing, and trapping, not only demand professional fishing skills and taxonomic knowledge of fish but also entail significant human and material resources and are detrimental to the river ecosystem (Evans and Lamberti 2018; Valentini et al. 2016; Yamamoto et al. 2016). Additionally, for fish residing in complex habitats, traditional methods often prove challenging to capture, leading to inaccuracies. To address the requirements of monitoring fish distribution under diverse environmental circumstances, it is essential to explore and develop a more comprehensive and efficient survey methodology. Environmental DNA (eDNA) refers to the totality of DNA fragments that can be directly extracted from environmental samples such as water, soil, air, ice cores, and the like (Stoeckle et al. 2017; Tillotson et al. 2018). DNA fragments in tissue detritus shed by organisms during their lifespan persist in the environment for a certain period (Tillotson et al. 2018). The eDNA detection approach is grounded on the specific gene recognition fragments of the investigated species or population and employs diverse molecular means to detect the recognition fragments contained in the DNA extracted from the environmental media, thereby ascertaining the distribution of organisms in the sampling environment (Bohmann et al. 2014; Taberlet et al. 2018). Some studies have shown that the eDNA technology can estimate species biomass and abundance based on the concentration of eDNA in water (Pilliod et al. 2013; Thomsen et al. 2012). The experiments of Thomsen et al. revealed a positive correlation between abundance and biomass and eDNA concentration, and suggested the use of eDNA methods as a biomonitoring tool. In recent years, the eDNA detection method has been widely favored in the domains of fish community assessment and ecology due to its advantages of high sensitivity, high efficiency, and noninvasiveness (Bohmann et al. 2014; R. Cheng et al. 2023, 2024; Doi et al. 2017).

The Xijiang River, the principal stream of the Pearl River Basin, is the third largest river in China, spanning a length of 2214 km. The Xijiang River boasts a long history, and each segment of the main stream has borne different names throughout history. The section from the source to Wangmo County in Guizhou Province is termed the Nanpan River, and the subsequent stretch to Xiangzhou County in Laibin City, Guangxi, is known as the Hongshui River. The Beipanjiang River is the main tributary of the Hongshui River. Additionally, the basin features highly developed karst landforms (Wang 2024; Yu 2006). According to records, the fishery resources in the Xijiang River are abundant, distributing a variety of rare and distinctive fish species. In the early 1980s, the Zhujiang Fisheries Research Institute and other units carried out a systematic survey of Xijiang's fishery resources and documented 136 species of fish (Lu 1990; Zheng 1989). Due to factors such as hydraulic construction, excessive fishing, water pollution, the number of fish populations in the Xijiang River has decreased significantly (Li et al. 2010).

In this study, the eDNA approach was employed to investigate the alterations of fish communities and their reactions to variations in hydrological conditions in the Xijiang main stream (Nanpanjiang River and Hongshui River) and its principal tributaries (Beipanjiang River) in Guizhou. Additionally, the influences of environmental factors on the composition of fish at various locations were explored, as well as the similarities and dissimilarities in fish diversity at these sections. The objective was to offer some specific recommendations for the conservation of Xijiang fish diversity.

## 2 Materials and Methods

### 2.1 The Study Area

**FIGURE 1:**
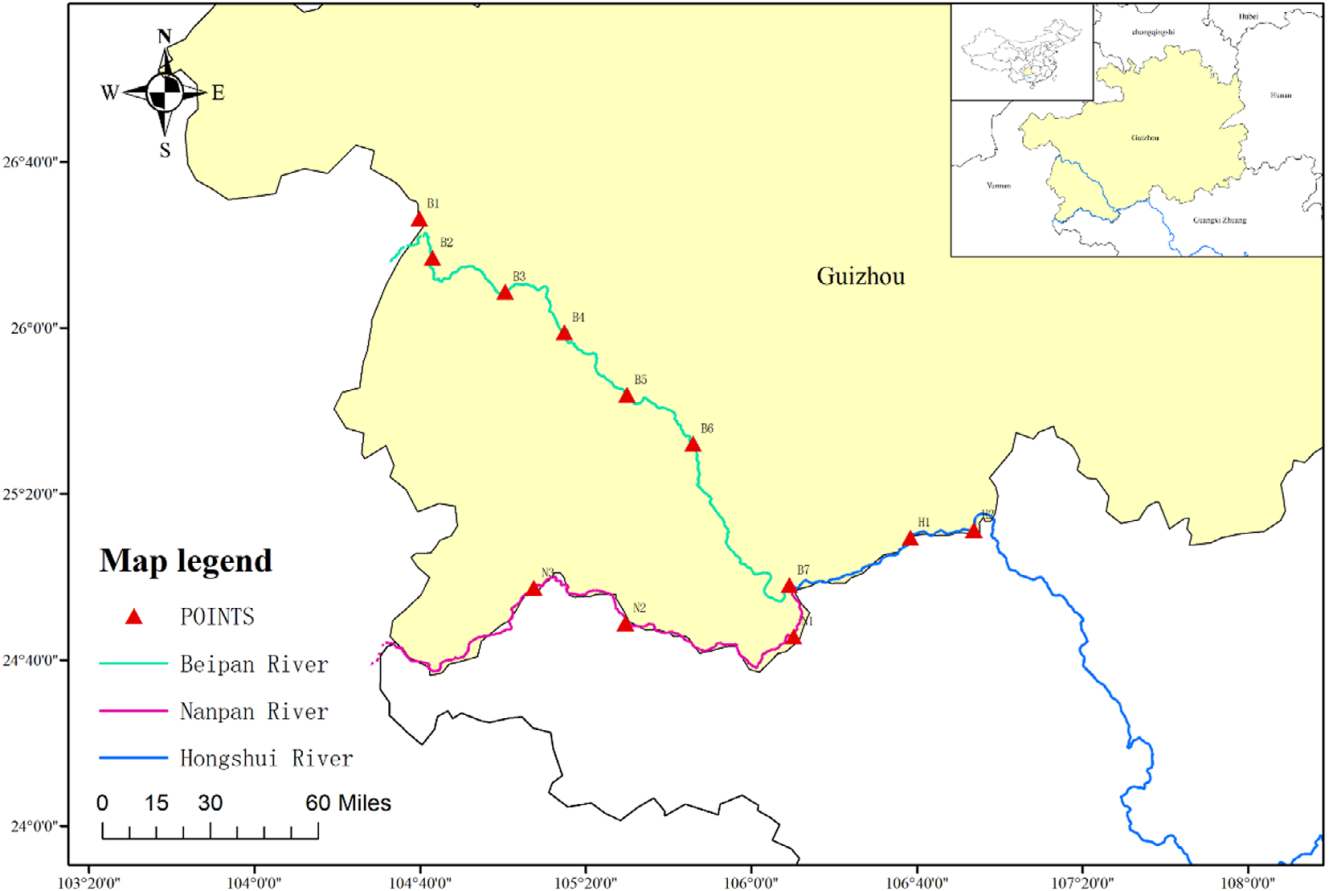
Map the geographic locations of sample sites using ArcMap 10.8 software (ESRI Inc., Redlands, CA, USA).

Considering the characteristics of the Xijiang aquatic ecosystem, 12 survey sites were selected in the Nanpanjiang, Hongshui, and Beipanjiang basins in Guizhou. Among them, the main tributary Beipanjiang River has seven stations, and the main streams Nanpanjiang River and Hongshui River have three and two survey sites, respectively. The sampling point B7 is the confluence of the three rivers (Figure 1). Detailed information regarding the sampling points is presented in Table 1.

**TABLE 1:** Sampling point information and environmental factors.

Sample	Longitude	Latitude	Temperature (°C)	pH	NH3‐N (mg/L)	DO (mg/L)	NO_2_ (mg/L)	PO4‐3 (mg/L)	Altitude (m)
SB1	104.6625	26.43786	26	8.6	0.25	9.6	0.013	0.08	1096.67
WB1	104.6625	26.43786	16.1	8.53	0.1	8.6	0.002	0.01	1096.67
SB2	104.7159	26.28018	29.2	8.8	0.12	10	0.01	0.04	870.57
WB2	104.7159	26.28018	17.2	8.37	0.1	8	0.006	0.01	870.57
SB3	105.0086	26.14388	27.4	8.75	0.11	10	0.018	0.05	696.25
WB3	105.0086	26.14388	21.8	8.39	0.1	9.2	0.009	0.01	696.25
SB4	105.2465	25.98099	26.5	8.75	0.12	7.9	0.007	0.05	696.13
WB4	105.2465	25.98099	20.4	8.25	0.1	8.2	0.002	0.01	696.13
SB5	105.4976	25.73041	23.7	8.71	0.1	8.5	0.002	0.02	559.01
WB5	105.4976	25.73041	20.5	8.15	0.11	8.5	0.017	0.01	559.01
SB6	105.7643	25.53365	28.3	8.62	0.1	9.1	0.002	0.01	461.01
WB6	105.7643	25.53365	23.1	8.31	0.1	9.4	0.004	0.01	461.01
SB7	106.1519	24.96627	29.6	8.49	0.1	10	0.007	0.03	311.84
WB7	106.1519	24.96627	23.6	8.3	0.11	7.8	0.003	0.01	311.84
SN1	106.1692	24.75939	25.6	8.31	0.25	8.9	0.004	0.02	330.7
WN1	106.1692	24.75939	21.3	8.39	0.37	8.7	0.012	0.01	330.7
SN2	105.4921	24.81342	21.5	8.07	0.1	7.1	0.002	0.01	382.5
WN2	105.4921	24.81342	21.7	8.18	0.11	9.2	0.016	0.01	382.5
SN3	105.1237	24.95461	24.1	8.06	0.1	6	0.009	0.01	620.68
WN3	105.1237	24.95461	22.6	8.12	0.11	7.8	0.005	0.01	620.68
SH1	106.6392	25.1565	30.2	8.55	0.19	10	0.011	0.07	341.83
WH1	106.6392	25.1565	25.8	8.23	0.15	8.4	0.005	0.02	341.83
SH2	106.8943	25.18773	29.2	8.53	0.1	9.1	0.012	0.01	334.87
WH2	106.8943	25.18773	24.7	8.02	0.15	7.4	0.002	0.01	334.87

### 2.2 Sampling and Environmental Factor Measurement

The water samples were collected in 2023 during the wet season (May 20th, represented by the capital letter, S) and the dry season (December 18th, represented by the capital letter, W). At each station, a 5 L‐capacity water sampler was utilized to collect water from the upper, middle, and lower levels of the river, and a representative 15 L mixed water sample was obtained after blending. The 3 L water sample was evenly divided into three 1 L aliquots, and the surplus water samples were discarded. Before each sampling, the sampling equipment was sterilized with a 10% bleach solution (Pilliod et al. 2013). A total of 72 samples were collected in two seasons. The collected samples were stored at low temperature and transported to the laboratory within 24 h. In the laboratory, the samples were concentrated on a 0.45 μm mixed cellulose membrane (hydrophilic filter membrane) through a vacuum pumping filter and finally frozen and stored in liquid nitrogen (−196°C) for DNA extraction. All equipment was disinfected during the operation to prevent cross‐contamination between samples. To eliminate contamination interference during the experimental process, this study established a full‐process negative control at both the sample collection and processing stages. The specific procedures are as follows: During each sampling event, an equal volume of pure water is taken as the negative control sample. The collection and preservation process for this pure water sample is identical to that of the environmental water sample, including the use of sterilized sampling equipment, transportation at low temperatures, and laboratory processing.

During the survey, altitude was ascertained through GPS location data. The portable water quality detector manufactured by PanTian Bio‐Tech was used to measure the water quality parameters on site. The parameters measured encompassed *T*, *DO*, *pH*, *NH*_*3*_ *‐N*, *NO*_*2*_^−^, and *PO*_*4*_^*3*−^.

### 2.3 eDNA Extraction and PCR Amplification

eDNA was extracted from water samples using the Omega Bio‐tek E.Z.N.A. Soil DNA Kit, following the manufacturer's protocol. To avoid contamination, extraction was carried out independently for each sample. The mitochondrial gene 12S rRNA, the most widely used molecular marker in fish diversity monitoring, was used as the target in amplification (Shu et al. 2020). The fish‐specific primer “Tele02” (Tele02‐F: 5′‐AAACTCGTGCCAGCCACC‐3′, Tele02‐R: 5′ GGGTATCTAATCCCAGTTTG‐3′), which was developed by Taberlet et al. (2018), was used in amplification with the addition of the sample‐specific barcode sequence. The PCR reactions were prepared in total volumes of 20 μL as follows: 4 μL 5× FastPfu Buffer, 2 μL dNTPs, 0.4 μL FastPfu Polymerase, 0.8 μL of each 10 μM primer, 2–5 μL of genomic DNA (10 ng/μL).

Finally, ddH_2_O was added to a total system volume of 20 μL. The PCR conditions included an initial denaturation step at 95°C for 5 min, followed by 27 cycles at 95°C for 30 s, 55°C for 30 s, and 72°C for 45 s, with a final duration of 10 min at 72°C. The products of PCR were sent to the Biozeron Company for sequencing.

### 2.4 eDNA Sequencing and Bioinformatics Processing

Samples were sequenced, and 250 bp paired‐end readings were obtained using a MiSeq sequencer (Illumina Inc., San Diego, CA, USA). Sequence data were sorted for each sample using the indices. Filter the base of the reads tail mass value below 20; set a window of 10 bp. If the average mass value in the window is lower than 20, cut off the back‐end base from the window; filter the reads below 50 bp after quality control. According to the overlap between PE reads, pairs of reads were merged into a sequence with a minimum overlap length of 10 bp. The overlap area of the splicing sequence allows the maximum mismatch ratio to be 0.2, and the nonconforming sequence is screened out. The samples were differentiated according to barcode and primer at both ends of the sequence, and the sequence direction was adjusted. The allowable mismatch number of the barcode was 0, and the maximum mismatch number of the primer was 2. Using Usearch software and the gold database, de novo and reference were used to remove chimeras. Singletons are removed during the OTU clustering process. The quality of the data from the readings was controlled, and the results were filtered using Trimmomatic v.0.36 (parameters—leading: 0; trailing: 20; sliding window: 10/20; and minlen: 75) (Bolger et al. 2014). Finally, the high‐quality sequences of each sample were retained by splitting the barcode and primer sequences using cut adapt with default parameters, and the sequence direction was corrected according to the positive and negative barcode and primer directions during the process. Paired reads were merged. Operational taxonomic unit (OTU) clustering analysis was performed according to a sequence similarity threshold of ≥ 97%, and representative OTU sequences were compared with sequences in the MitoFish “http://mitofish.aori.u‐tokyo.ac.jp” and NCBI “https://www.ncbi.nlm.nih.gov” databases for classification annotation and generation of the corresponding OTU abundance table. Sequences of unmatched species were culled from the samples at each sampling point, and the sequences of matched species were averaged. After removing the sequence data showing high identity to nonfish organisms (such as bacteria, birds, amphibians, mammals, and so forth.), the remaining filtered data were compared with fish sequences and showed ≥ 97% identity and *E*‐values ≤ 10, and OTUs corresponding to the same species were merged. If any OTU did not show adequate similarity for identification at the species level, statistical analyses were then carried out for a higher taxonomic level such as genus and family. The sequence number proportion of each species in each sample was calculated in Excel. The taxonomic information of fish was improved by referring to the Fishbase database “https://www.fishbase.in/home.htm”.

### 2.5 Fish Diversity Analysis

The Alpha diversity analysis of each sample can reflect the abundance and diversity of environmental biomes. In this study, the *Shannon* index (Shannon 1948), *Simpson* index (Simpson 1949) and *Pielou* index (Pielou 1966) were selected to reflect community richness and community diversity.

To identify potential principal components that affect differences in community composition in the sample by reducing dimensions; nonmetric multidimensional scaling (NMDS) analysis of community compositions at different sample points was based on the Bray–Curtis distance algorithm to explore differences or similarities in community composition among different groups of samples (NMDS uses the ape package and the ggplot2 package).

### 2.6 Environmental Factors Correlation Analysis

In this study, redundancy analysis (RDA) was selected to reflect the relationship between fish communities and environmental factors. Elevation, *T*, *DO*, *pH*, *NH3‐N*, *NO*_*2*_^−^, and *PO*_*4*_^*3*−^ were grouped by season for analysis. The RDA data were standardized by Hellinger in the R software, then calculated using the vegan package, and finally presented graphically in the ggplot2 package (Legendre et al. 2011).

### 2.7 Collection of Historical Fish Data

Based on the literature “Fishes of Guizhou” and “Fishery Resources of the Pearl River System,” along with recent research on Xijiang fish stocks over the past 2 decades, we have compiled a comprehensive historical inventory of fish species in the Guizhou section of the Xijiang River.

## 3 Results

### 3.1 Fish Community Structure Composition

In the study area, a total of 72 samples were collected during the wet season and dry season in 2023. All samples were successfully amplified after quality inspection and underwent high‐throughput sequencing. Following quality control and filtration as outlined in Section 2.4 of the bioinformatics processing protocol, we identified a total of 9,025,640 sequences corresponding to 97 fish species across 12 orders, 27 families, and 74 genera through BLAST analysis and manual correction. During the wet season, we detected 65 fish species distributed among 11 orders, 22 families, and 54 genera; whereas in the dry season, we identified a total of 71 fish species across 10 orders, 23 families, and 56 genera.

The results indicate that Cypriniformes had a significant advantage, accounting for 70.1% (*N* = 68); followed by Siluriformes accounting for 11.34% (*N* = 11). At the family level, Cyprinidae had the largest number of fish with 51.55%; followed by Xenocyprididae with 8.25% (Figure S1). *Rhinogobius giurinus* , *Hemiculter leucisculus* , *Culter alburnus* , *Coptodon zillii*, and *Hypophthalmichthys nobilis* dominated in sequence abundance (Figure S2).

### 3.2 Compare Historical Data

In light of the “Fishes of Guizhou” and “Fishery Resources of the Pearl River System,” with recent research on fish communities in the Xijiang River, we have compiled a comprehensive historical inventory of fish species relevant to the Guizhou segment of the Xijiang River Basin, includes 134 species of fish (Table 2) (Lu 1990; Wang et al. 2019; Wu 1987). eDNA survey results indicate that 83 species on the list were detected, with a detection rate of 61.94%. Four families, Centrarchidae, Engraulidae, Gobionidae, and Tetraodontidae, appear only in eDNA results, while two families, Poeciliidae and Synbranchidae, appear only in historical data. The eDNA analysis identified 14 new fish species, including *Micropterus salmoides* , *Coptodon zillii*, *Oreochromis mossambicus* , *Oreochromis niloticus* , and *Parachromis managuensis* —five of which are recognized as typical invasive species. Notably, *Coptodon zillii* emerged as one of the five most abundant fish in the findings (Figure S2), suggesting an increasing proportion of exotic fish within the ecosystem.

**TABLE 2:** Fish list based on eDNA metabarcoding and historical data.

Species	Habitat	Record	(S)	(W)
**Acipenseriformes**				
Acipenseridae				
*Acipenser baerii*	Demersal	+		+
**Anguilliformes**				
Anguillidae				
*Anguilla japonica*	Demersal	+	+	+
**Cypriniformes**				
Cyprinidae				
*Platysmacheilus nudiventris*	Benthopelagic		+	
Cyprinidae				
*Abbottina rivularis*	Benthopelagic	+	+	+
*Acheilognathus barbatus*	Benthopelagic	+	+	
*Acheilognathus macropterus*	Benthopelagic	+		
*Acheilognathus tonkinensis*	Benthopelagic	+	+	
*Acrossocheilus clivosius*	Benthopelagic	+		
*Acrossocheilus longipinnis*	Benthopelagic	+		
*Acrossocheilus parallens*	Benthopelagic	+	+	
*Acrossocheilus yunnanensis*	Benthopelagic	+	+	+
*Hypophthalmichthys nobilis*	Benthopelagic	+	+	+
*Bangana decora*	Benthopelagic	+		
*Bangana wui*	Benthopelagic	+		
*Bangana zhui*	Benthopelagic	+		
*Carassius auratus*	Benthopelagic	+	+	+
*Ctenopharyngodon idella*	Benthopelagic	+		+
*Culter alburnus*	Benthopelagic	+	+	+
*Culter recurviceps*	Benthopelagic	+		
*Cyprinus carpio*	Benthopelagic	+	+	+
*Discogobio brachyphysallidos*	Benthopelagic	+	+	+
*Discogobio laticeps*	Benthopelagic	+		+
*Discogobio multilineatus*	Benthopelagic	+		
*Discogobio tetrabarbatus*	Benthopelagic	+		
*Discogobio yunnanensis*	Benthopelagic	+	+	+
*Elopichthys bambusa*	Benthopelagic	+		
*Foliter brevifilis*	Benthopelagic	+		+
*Garra imberba*	Benthopelagic	+		
*Garra orientalis*	Benthopelagic	+		
*Hemibarbus labeo*	Benthopelagic	+		+
*Hemibarbus maculatus*	Benthopelagic	+	+	+
*Hemiculter leucisculus*	Benthopelagic	+	+	+
*Hypophthalmichthys molitrix*	Benthopelagic	+	+	+
*Linichthys laticeps*	Benthopelagic	+		
*Luciocyprinus langsoni*	Benthopelagic	+	+	
*Metzia lineata*	Benthopelagic	+		
*Mylopharyngodon piceus*	Demersal	+	+	
*Ochetobius elongatus*	Benthopelagic	+	+	+
*Onychostoma gerlachi*	Benthopelagic	+	+	
*Onychostoma ovalis*	Benthopelagic	+		+
*Opsariichthys bidens*	Benthopelagic	+	+	+
*Parabramis pekinensis*	Benthopelagic	+		+
*Parator zonatus*	Benthopelagic	+		
*Percocypris pingi*	Benthopelagic	+		
*Percocypris pingi regani*	Benthopelagic	+		
*Procypris merus*	Benthopelagic	+	+	+
*Pseudocrossocheilus bamaensis*	Benthopelagic	+		
*Pseudogyrincheilus prochilus*	Benthopelagic	+		
*Pseudohemiculter dispar*	Benthopelagic	+	+	+
*Pseudorasbora parva*	Benthopelagic	+		+
*Ptychidio jordani*	Demersal	+	+	
*Ptychidio macrops*	Benthopelagic	+		
*Puntius semifasciolatus*	Benthopelagic	+	+	
*Qianlabeo striatus*	Benthopelagic	+		
*Rhodeus ocellatus*	Benthopelagic	+	+	+
*Sarcocheilichthys parvus*	Benthopelagic	+	+	
*Sarcocheilichthys sinensis*	Benthopelagic	+		
*Saurogobio dabryi*	Benthopelagic	+		
*Schizothorax grahami*	Benthopelagic	+		
*Schizothorax griseus*	Benthopelagic	+		+
*Schizothorax lissolabiatus*	Benthopelagic	+		
*Semilabeo notabilis*	Benthopelagic	+	+	
*Semilabeo obscurus*	Benthopelagic	+	+	
*Sinibrama macrops*	Benthopelagic	+		
*Sinocyclocheilus angustiporus*	Benthopelagic	+		
*Sinocyclocheilus bicornutus*	Benthopelagic	+		
*Sinocyclocheilus cyphotergous*	Benthopelagic	+		
*Sinocyclocheilus lateristritus*	Benthopelagic	+		
*Sinocyclocheilus multipunctatus*	Benthopelagic	+		
*Sinocyclocheilus oxycephalus*	Benthopelagic	+		
*Sinocyclocheilus robutus*	Benthopelagic	+		
*Spinibarbus denticulatus denticulatus*	Benthopelagic	+	+	+
*Spinibarbus denticulatus polylepis*	Benthopelagic	+		
*Spinibarbus hollandi*	Benthopelagic	+	+	+
*Squalidus argentatus*	Benthopelagic	+	+	+
*Squaliobarbus curriculus*	Benthopelagic	+	+	+
*Zacco platypus*	Benthopelagic	+		+
*Discogobio macrophysallidos*	Benthopelagic	+		+
*Anabarilius duoyiheensis*	Benthopelagic	+	+	
*Anabarilius liui*	Benthopelagic	+		+
*Aphyocypris chinensis*	Benthopelagic	+	+	
*Carassioides acuminatus*	Benthopelagic		+	
*Carassius cuvieri*	Demersal	+		+
*Cirrhinus molitorella*	Benthopelagic	+	+	+
*Discocheilus wui*	Benthopelagic			+
*Hemiculterella wui*	Benthopelagic	+	+	+
*Megalobrama amblycephala*	Benthopelagic	+		+
*Megalobrama terminalis*	Benthopelagic	+	+	
*Onychostoma simum*	Benthopelagic	+	+	+
*Osteochilus salsburyi*	Benthopelagic	+	+	+
*Pseudocrossocheilus tridentis*	Benthopelagic		+	
*Sinocrossocheilus bamaensis*	Benthopelagic			+
*Sinocyclocheilus rhinocerous*	Benthopelagic	+		+
*Toxabramis houdemeri*	Benthopelagic	+	+	+
*Xenocypris argentea*	Benthopelagic	+	+	
Balitoridae				
*Sinogastromyzon sichangensis*	Demersal			+
*Beaufortia huangguoshuensis*	Benthopelagic	+		
*Beaufortia kweichowensis*	Demersal	+	+	
*Beaufortia pingi*	Benthopelagic	+		
*Hemimyzon macroptera*	Benthopelagic	+		
*Hemimyzon pumilicorpora*	Benthopelagic	+		
*Paraprotomyzon multifasciatus*	Benthopelagic	+		
*Sinogastromyzon wui*	Benthopelagic	+		
*Vanmanenia pingchowensis*	Benthopelagic	+		+
Cobitidae				
*Paramisgurnus dabryanus*	Demersal	+	+	+
Nemacheilidae				
*Misgurnus anguillicaudatus*	Demersal	+	+	+
*Parabotia fasciata*	Demersal	+		+
*Homatula variegatus*	Benthopelagic	+		
*Schistura incerta*	Benthopelagic	+		
*Triplophysa zhenfengensis*	Benthopelagic	+		
*Schistura fasciolata*	Benthopelagic	+	+	+
Botiidae				
*Sinibotia robusta*	Demersal	+		+
Cyprinidae				
*Rhodeus sinensis*	Benthopelagic			+
**Siluriformes**				
Sisoridae				
*Glyptothorax fukiensis*	Benthopelagic	+		
*Pareuchiloglanis feae*	Benthopelagic	+		
*Pareuchiloglanis longicauda*	Benthopelagic	+		+
Siluridae				
*Pterocryptis cochinchinensis*	Benthopelagic	+		
*Silurus asotus*	Demersal	+	+	+
*Silurus meridionalis*	Demersal	+	+	+
*Silurus grahami*	Demersal	+	+	
Loricariidae				
*Hypostomus plecostomus*	Demersal	+	+	
Clariidae				
*Clarias gariepinus*	Benthopelagic	+	+	+
Bagridae				
*Hemibagrus guttatus*	Benthopelagic	+		
*Hemibagrus macropterus*	Demersal	+		+
*Pelteobagrus fulvidraco*	Demersal	+		+
*Pelteobagrus vachelli*	Demersal	+	+	+
*Pseudobagrus albomarginatus*	Benthopelagic	+		
*Leiocassis longirostris*	Demersal	+	+	
Ictaluridae				
*Ictalurus punctatus*	Demersal	+	+	+
**Cyprinodontiformes**				
Poeciliidae				
*Gambusia affinis*	Benthopelagic	+		
Cyprinodontidae				
*Oryzias sinensis*	Benthopelagic	+	+	
**Perciformes**				
Gobiidae				
*Rhinogobius giurinus*	Demersal	+		+
*Rhinogobius brunneus*	Demersal	+		+
*Rhinogobius cliffordpopei*	Benthopelagic	+		+
Eleotridae				
*Eleotris oxycephala*	Demersal	+	+	
Centrarchidae				
*Micropterus salmoides*	Benthopelagic			+
Percichthyidae				
*Siniperca kneri*	Benthopelagic	+	+	+
*Siniperca scherzeri*	Benthopelagic	+		
Cichlidae				
*Coptodon zillii*	Benthopelagic			+
*Oreochromis mossambicus*	Benthopelagic		+	+
*Oreochromis niloticus*	Benthopelagic		+	+
*Parachromis managuensis*	Benthopelagic			+
Channidae				
*Channa asiatica*	Benthopelagic	+	+	
*Channa argus*	Benthopelagic	+	+	+
**Synbranchiformes**				
Mastacembelidae				
*Mastacembelus armatus*	Demersal	+	+	+
Synbranchidae				
*Monopterus albus*	Benthopelagic	+		
**Clupeiformes**				
Engraulidae				
*Coilia brachygnathus*	Pelagic			+
**Tetraodontiformes**				
Tetraodontidae				
*Takifugu ocellatus*	Benthopelagic		+	

### 3.3 Spatial Patterns of Community Structure Composition and Species Diversity Analysis

**FIGURE 2:**
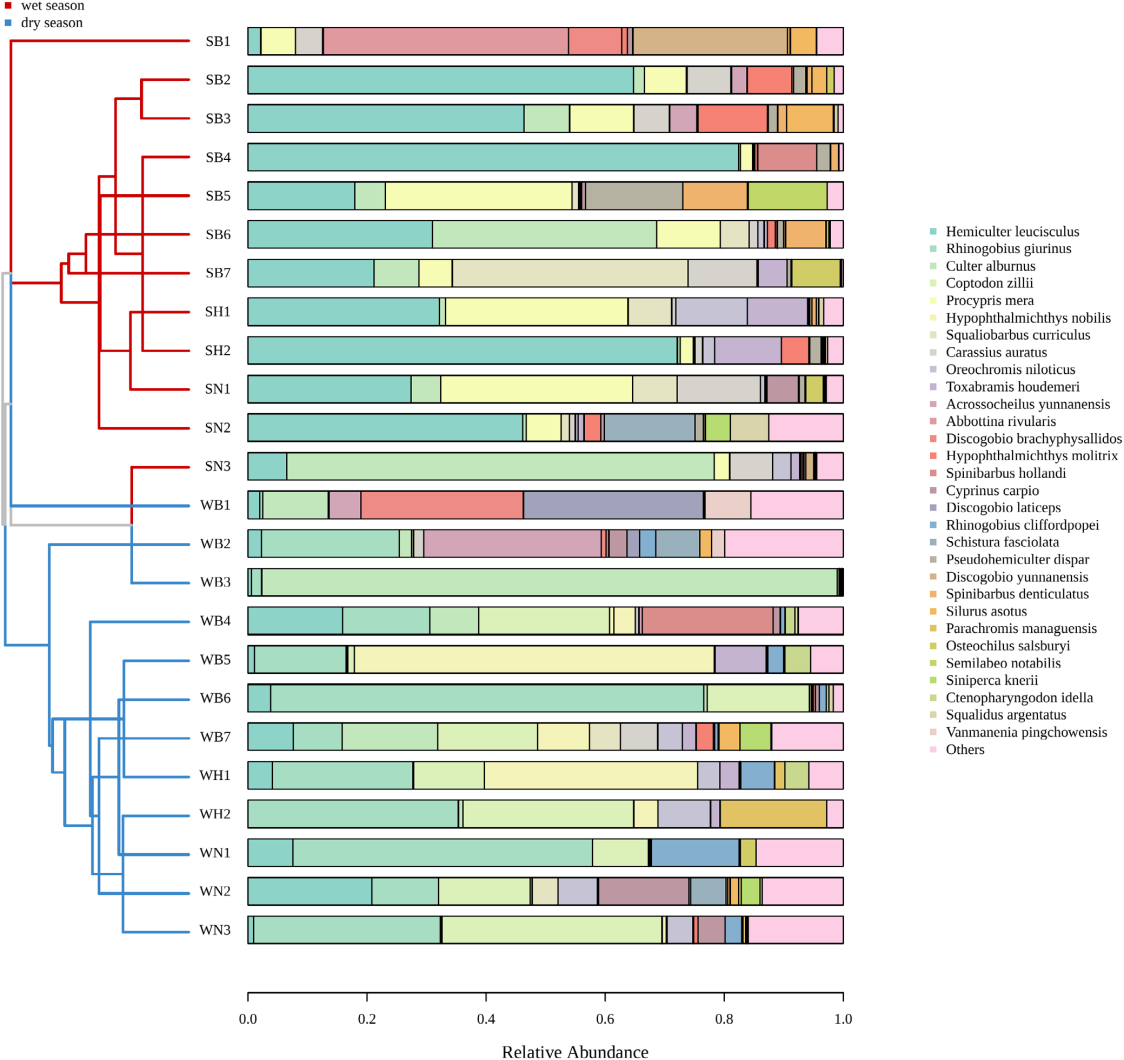
Cluster tree stack histogram based on fish abundance at each point.

**FIGURE 3:**
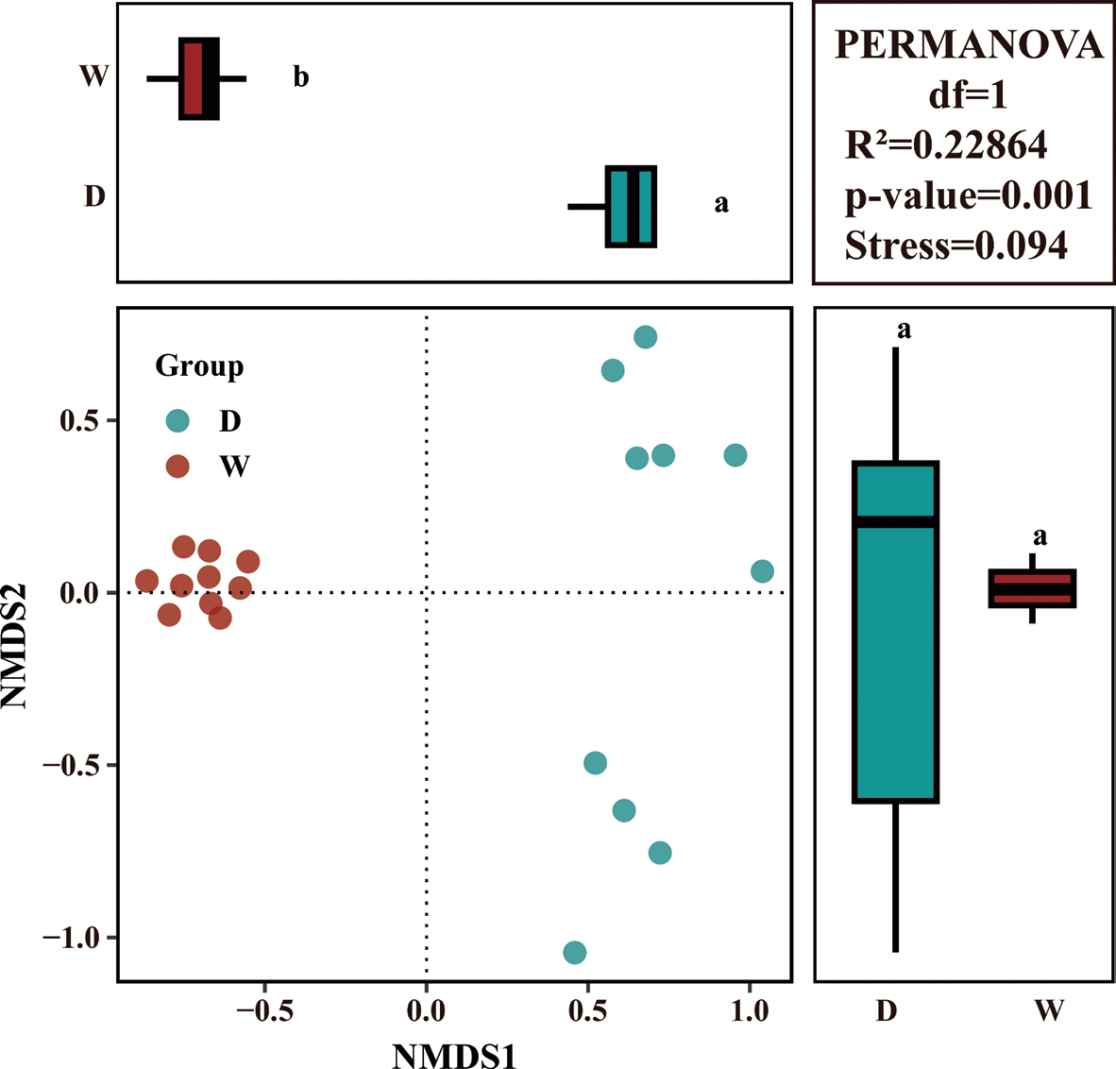
Fish species detection: Intergroup differences in nonmetric multidimensional scaling (NMDS).

**FIGURE 4:**
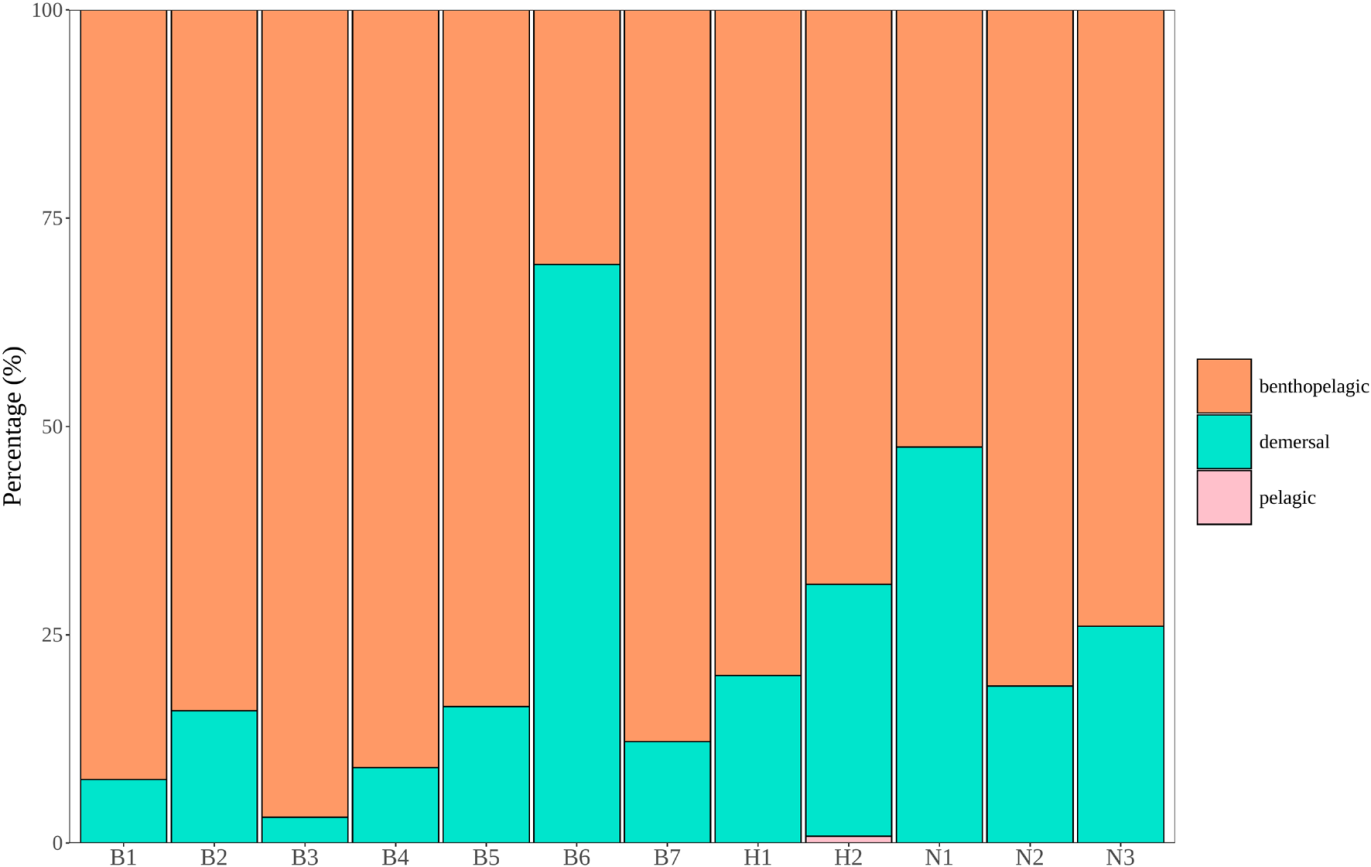
Habitat water layer based on relative sequences of fish at each point represented by the bar chart.

Regarding fish composition, significant differences in fish communities between the two seasons were observed in the Xijiang River Basin of Guizhou Province. Furthermore, there exist differences among each point (Figure 2). This was also confirmed by nonmetric multidimensional scaling (NMDS) results based on fish abundance (Figure 3). We then conducted a SIMPER (Similarity Percentages) Analysis and found that *Rhinogobius giurinus* was the species that contributed the most to the differences between seasons (Table S1). Furthermore, the fish composition across various habitat water layers at most sites exhibited a high degree of consistency, with benthopelagic fish predominating. In contrast, B6 sites displayed notable differences, where demersal fish emerged as the dominant type (Figure 4). The results of the Alpha diversity analysis indicated that the *Shannon* index, *Simpson* index, and *Pielou* index exhibited similar trends across all sampling points, with the WN2 station recording the highest values and the WB3 station displaying the lowest. The *Shannon* index ranged from 0.2 to 2.5, while the *Simpson* index varied between 0.06 and 0.88, and the *Pielou* index spanned from 0.06 to 0.72 (Table 3). Nevertheless, significant differences were observed among the sampling points (*p* < 0.05) (Figure S3).

**TABLE 3:** Alpha diversity synthesis analysis.

Sample point	SB1	SB2	SB3	SB4	SB5	SB6	SB7	SH1	SH2	SN1	SN2	SN3
Shannon	1.6967	1.1551	1.5533	0.8338	1.7916	1.6391	0.7853	1.6971	1.1054	1.4196	1.6750	1.0954
Simpson	0.7350	0.4804	0.6613	0.3840	0.7933	0.7118	0.3312	0.7547	0.4522	0.5709	0.6739	0.4329
Pielou_J	0.5898	0.3992	0.5283	0.2755	0.6142	0.5957	0.2749	0.5963	0.3447	0.4462	0.5490	0.3442

Sample point	WB1	WB2	WB3	WB4	WB5	WB6	WB7	WH1	WH2	WN1	WN2	WN3
Shannon	1.7496	2.0737	0.2005	2.0019	0.9873	0.9127	1.9727	1.3373	1.4333	1.3326	2.4280	1.8141
Simpson	0.7772	0.8084	0.0644	0.8294	0.4466	0.4316	0.8141	0.5828	0.7182	0.6537	0.8744	0.7750
Pielou_J	0.5422	0.6967	0.0656	0.6361	0.3335	0.3058	0.6295	0.5242	0.5924	0.4775	0.7146	0.6383

### 3.4 Responses of Fish Communities to Environmental Factors

**FIGURE 5:**
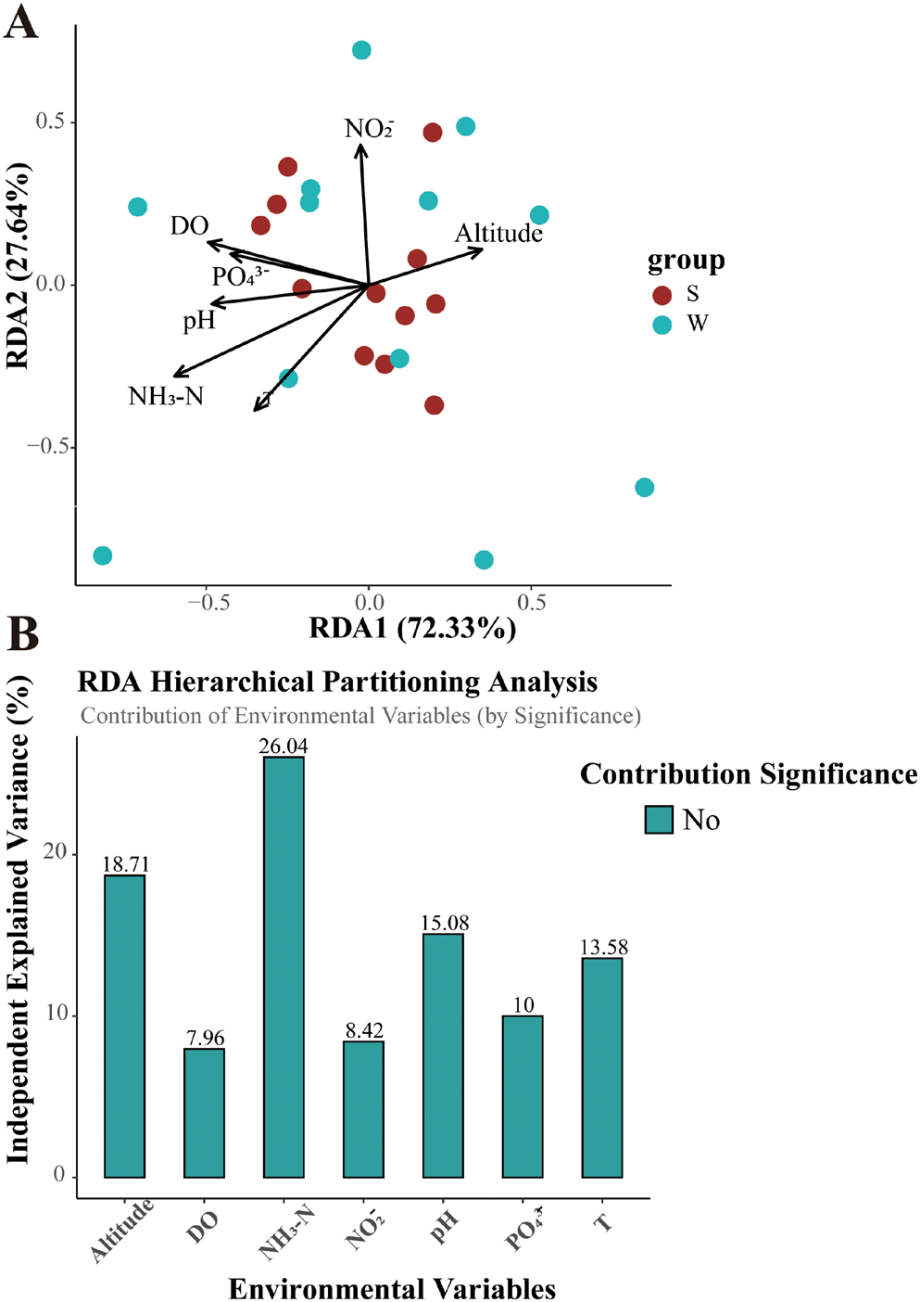
RDA showing the correlation between environmental factors and fish communities.

To comprehend the causes of the diversity of fish communities in various regions of the Xijiang River Basin in Guizhou Province, redundancy analysis (RDA) was employed to analyze the environmental factors influencing the distribution of fish communities (Figure 5). The first axis (RDA1) accounts for 72.33% of the variance, and the second axis (RDA2) accounts for 27.64% of the variance. The results indicated that the environmental factors influencing fish community structure in the Xijiang River Basin, in descending order of impact, were *NH*_*3*_ *‐N*, *Altitude*, *pH*, *T*, *PO*_*4*_^*3*−^, *NO*_*2*_^−^, and *DO*. The differences in fish community structure were primarily influenced by *NH*_*3*_ *‐N* (26.04%), *Altitude* (18.71%), and *pH* (15.08%), which aligns with the findings of the α‐diversity analysis. Detailed environment factor parameters are shown in Table 1.

## 4 Discussion

### 4.1 The Fish Community Demonstrated a Trend of Miniaturization, Which Was Accompanied by an Increase in the Prevalence of Non‐Native Species

The results demonstrated that the fish population structure in the Xijiang River basin of the Guizhou section has changed. The number of rare endemic fish, migratory fish, and rapids fish has decreased, and exotic fish have gradually become the dominant species, which is in line with the outcomes of many other fish studies in the Xijiang River (Gu et al. 2022; Wang et al. 2019; Zhu et al. 2022). The influence of reservoir construction on fish diversity and community structure is inevitable, and numerous reports have recorded the disappearance of fish species after reservoir construction (Gao et al. 2010; Santos et al. 2017). The construction of the reservoir broadened the surface and increased the capacity of the river, but it also led to a reduction in the water flow. This might well explain why some previously abundant rapids fish, including *Paraprotomyzon multifasciatus* , *Parator zonatus* , and *Percocypris pingi* , were not observed in this study. Notably, *Parator zonatus* is a vulnerable species, and *Percocypris pingi* is an endangered species. The formation of reservoirs can cause the deposition of organic matter and affect the scouring of river channels by water currents, which in turn can bring about changes in the structure of bait organisms and ultimately influence the composition of fish communities (Cao 2017). In conclusion, the Xijiang River basin in the Guizhou section is currently characterized by a prevalence of benthopelagic fish.

In this study, we discovered that *Rhinogobius giurinus* was the predominant species with a high relative sequence abundance in most sampling sites. Additionally, the dominant species at each sampling site mainly consisted of smaller species like *Rhinogobius giurinus, Hemiculter leucisculus*, and *Coptodon zillii*, as well as omnivorous species such as *Hypophthalmichthys nobilis* . Similar results have been shown in previous studies of traditional investigation methods for different sections of the Xijiang River (Liu et al. 2024). For example, Feng et al. demonstrated that fish in the Laibin section of the Xijiang River were predominantly small and omnivorous (Feng et al. 2022). Additionally, Wang et al. investigated the fish resources in the main stream of the Hongshui River and found that small‐sized species constituted the majority of the catch in terms of quantity (Chong et al. 2015). In conclusion, a discernible trend of species miniaturization and the prevalence of omnivorous species has been observed in the Guizhou section of the Xijiang River Basin. This trend may be attributed to the adaptability of smaller fish and omnivorous species, which benefit from abundant foraging space and food resources in the context of dam‐induced environmental changes (Hoeinghaus et al. 2009; Perera et al. 2014). This type of fish is highly adaptive to changes in the external environment and does not have strong migratory habits. Therefore, when the dam causes alterations in the river habitat, they can still survive normally and will not be strongly affected (Agostinho et al. 2008). For instance, *Rhinogobius giurinus* is a migratory fish but can also form landlocked populations with strong adaptability. At the same time, it may cause biological invasion and impose considerable pressure on the survival of indigenous fish by swallowing their eggs and competing with them for food and space. *Coptodon zillii* was introduced into China from Thailand in 1978. Due to its small individual size, slow growth rate, and other factors, it was quickly eliminated through breeding after introduction. However, its low temperature tolerance and strong fertility facilitated the invasion and diffusion of *Coptodon zillii* in natural waters.

Additionally, among the 14 fish species identified by eDNA analysis that are not listed in historical fish records, five species, including *Micropterus salmoides* , *Coptodon zillii*, *Oreochromis mossambicus* , *Oreochromis niloticus* , and *Parachromis managuensis* , are considered typical invasive species. Notably, *Coptodon zillii* is one of the five fish species with the most abundant sequence abundance, indicating that the proportion of exotic fish in the aquatic ecosystem of the Xijiang River Basin in Guizhou is increasing.

### 4.2 The Distribution of Species Within the Xijiang River Basin in Guizhou Province Was Characterized by Unevenness; the Community Structure Exhibited Instability

The structure of the fish community exhibited distinct compositions at each sampling point. Similar results were found in the NMDS analysis. The alpha diversity index of the different sampling points was significantly dissimilar, suggesting that the fish diversity within the basin was inconsistent. Additionally, the species diversity index serves as a metric for assessing the stability of community structure and its response, evaluated through two dimensions: the uniformity of the population and the number of individuals within the population (Santini et al. 2017). A higher diversity index, indicative of a greater number of species and a more uniform distribution of those species, correlates with increased community stability. Overall, the average α‐diversity index in the Xijiang River Basin of Guizhou Province exhibits a relatively low level with uneven distribution, suggesting an uneven distribution of species and an unstable community structure. This phenomenon may be related to habitat fragmentation, barrier formation, and river ecosystem degradation caused by dam construction in the Xijiang River Basin, Guizhou province, resulting in a decrease in the number of vulnerable native fish and a rapid increase in the number of adaptable exotic fish, further leading to biological homogeneity and a decrease in fish diversity (Liu et al. 2019; Petesse and Petrere 2012; Sá‐Oliveira et al. 2015). Collectively, these factors may have contributed to a reduction in fish diversity and variations in alpha diversity across different river reaches.

Water temperature significantly influences the metabolic processes of ectothermic animals, thereby affecting their growth (McKenzie 2023). Furthermore, water temperature plays a critical role in determining species distribution within ecological communities, with fluctuations in temperature prompting certain species to migrate to more favorable habitats (Baptista et al. 2019). In the Xijiang River basin in Guizhou, the average water temperature is higher during the wet season months compared with the dry season. However, the dry season average still reaches 21.6°C. Results from NMDS indicate notable differences in fish community structure between the two seasons. The alpha diversity index and species richness were observed to be lower in the wet season than in the dry season, a finding that contradicts the results reported by Chen et al. (Chen et al. 2024). Notably, we observed that the dry season temperatures in the Xijiang River basin are comparable to those recorded in autumn at the Yangtze River Estuary. Research indicates that excessive water temperatures can adversely affect the growth and reproduction of fish (Dahlke et al. 2020). In short, this phenomenon may be related to the higher water temperatures observed in the Xijiang River Basin in Guizhou in the wet season.

### 4.3 The Driving Effects of Altitude and Season on the Structure of Fish Communities

The effects of environmental factors on aquatic biodiversity have been extensively researched. Previous studies have indicated that fish diversity is typically influenced by diverse environmental factors, such as *NH*_*3*_ *‐N*, *T*, *DO*, *pH*, and the altitude of the catchment (Kouamélan et al. 2003; Jianhua Li et al. 2012; Wolter 2007). In this study, location and seasonal variation are the crucial factors affecting fish diversity, which has an impact on various environmental factors like *NH*_*3*_ *‐N* and *pH*. Cheng et al. discovered that water temperature exerts a significant effect on fish diversity, and the main mechanism is that it simultaneously affects the survival and reproduction of fish. The minimum temperature required for the development of many fish, especially those with drifting eggs, is 18°C. But the existence of reservoirs can cause a delay in the warming of water temperatures, and a delayed spawning season can shorten the growing season of young fish before the dry season, reducing their energy accumulation and thereby reducing their overdry seasoning survival rate (Cheng et al. 2015). This study has found that the water temperature in the Guizhou section of the Xijiang River basin was high, which was suitable for the growth of most fish. Despite the presence of the cascade dam, the water temperature has not been significantly affected. The average water temperature is 26.8°C in the wet season and 21.6°C in the dry season. This might be attributed to the unique climate characteristics of the area. DO reflects the self‐purification capacity of the water body; it is conducive to the growth of bait organisms in a high‐oxygen environment, providing sufficient food sources for fish and thus influencing the distribution of fish communities (Zhang et al. 2023).

Different elevations could result in variations in water *NH*_*3*_ *‐N*, *T*, *DO*, and other environmental factors, which would indirectly affect the diversity of fish. Studies have demonstrated that elevation plays a vital role in the formation of diversity in fish communities, and higher elevations restrict the abundance of fish species in rivers (Ding et al. 2012; Shu‐Li et al. 2022). By comparing two streams, Suarez et al. found that elevation is one of the most significant environmental factors affecting species diversity and abundance (Súarez et al. 2007). The results of the RDA analysis in this study indicate that altitude is significantly correlated with the structure of the fish community. This finding is consistent with the research conducted by Suarez et al. Furthermore, the difference in altitude between the highest and lowest sampling points exceeds 700 m, suggesting that altitude may be a critical factor influencing fish communities.

### 4.4 The Feasibility and Limitation of eDNA Method

As a novel biological detection technology, eDNA exerts a significant influence in the field of biological monitoring. However, it also presents numerous characteristics, encompassing both advantages and limitations (Deiner et al. 2015; S. Zhang et al. 2022). The eDNA method is highly sensitive and capable of detecting extremely low concentrations of biological DNA in the environment, enabling it to identify species that are rare and challenging to observe directly (Alexander et al. 2020; Jackman et al. 2021). For instance, in extreme environments like the deep sea or areas with concealed habitats, where traditional survey methods prove ineffective, eDNA technology can detect potential biological species, offering a novel approach for biological resource exploration (Miya 2022; Stat et al. 2017). Its noninvasive nature is another advantage. Unlike traditional investigation methods such as fishing, trapping, or direct observation, which might cause disturbances or harm to organisms, eDNA detection merely requires the collection of environmental samples, such as water bodies and soil, without directly damaging individual organisms and their habitats, thereby facilitating the research and protection of rare species and ecologically fragile areas (Zhang et al. 2022).

However, the eDNA approach is not without flaws. There are challenges regarding testing accuracy, with false‐positive and false‐negative results occurring from time to time. Bohmann et al. suggest that this might be attributed to the lack of specificity of the PCR primers employed (Bohmann et al. 2014). Therefore, the primer utilized in our study is “Tele02,” which is commonly used for freshwater fish. In species identification, complex factors in the environment may cause the DNA of nontarget species to be mistakenly detected as that of target species, thereby influencing the accurate determination of biological species. Before the bioanalysis commences, manual correction will be carried out to avoid such circumstances. At the same time, the dynamic change of eDNA also poses difficulties to the detection. Studies have indicated that the dispersal of fish eDNA in the lower reaches of rivers is generally maintained within a range of 2 km (Jane et al. 2015; Understanding environmental DNA detection probabilities Wilcox et al. 2016). Hence, to ensure the feasibility of the eDNA method, our study aimed to establish sampling sites within 2 km downstream of each reservoir and within 2 km downstream of the backwater bay.

## 5 Conclusions

The eDNA technology was employed to investigate the fish diversity in the Xijiang River Basin of Guizhou Province, disclosing the composition and diversity of the fish communities. The results indicated that Cyprinidae was the primary fish community in the Xijiang River Basin. Under the influence of human factors such as reservoirs, the composition of the fish community in the Xijiang River Basin in Guizhou has altered. There were notable differences in the composition of the fish community in different seasons. There were also disparities in fish diversity and composition at each sampling site. The fish populations demonstrated a trend of species miniaturization, and invasive species gradually became the dominant ones. This further resulted in the homogenization of the fish populations. The environmental factors influencing the structure of the fish community in the Xijiang River Basin, in descending order of importance, were *NH*_*3*_ *‐N*, *altitude*, *pH*, *T*, *PO*_*4*_^*3*−^, *NO*_*2*_^−^, and *DO*. Among these, *NH*_*3*_ *‐N*, *altitude*, and *pH* had a particularly significant impact on the community structure of fish. This research furnishes significant data and insights for comprehending the fish diversity in the Xijiang River Basin of Guizhou and aims to offer a scientific basis for future conservation and management endeavors.

## Author Contributions

**Xiuhui Ma:** conceptualization (equal), data curation (equal), funding acquisition (equal), methodology (equal), writing – original draft (equal), writing – review and editing (equal). **Fujiang Huang:** funding acquisition (equal), investigation (equal), methodology (equal), project administration (equal). **Ruiyuan Zhang:** formal analysis (equal), investigation (equal), software (equal), validation (equal). **Tianhong Liu:** investigation (equal). **Tianyang Zhang:** investigation (equal). **Peng Zeng:** formal analysis (equal), investigation (equal), validation (equal), writing – original draft (equal), writing – review and editing (equal).

## Consent

The authors have nothing to report.

## Conflicts of Interest

The authors declare no conflicts of interest.

## Supporting information


**Figure S1:** ece371825‐sup‐0001‐FigureS1.tif.

**Figure S2:** ece371825‐sup‐0002‐FigureS2.tif.

**Figure S3:** ece371825‐sup‐0003‐FigureS3.tif.

**Table S1:** ece371825‐sup‐0004‐TableS1.docx.

## Data Availability

The raw sequence data reported in this paper were submitted to the NCBI Sequence Read Archive (SRA) database (Accession No.: PRJNA1199918) https://dataview.ncbi.nlm.nih.gov/object/PRJNA1199918?reviewer=l910p5lkiara0iphciceb222lj.
