# Supplementary figures and images for: Environmental DNA Reveals the Fish Community Structure Exhibited Instability and Trend of Miniaturization in the Xijiang River Basin of the Guizhou
Source: Ecol Evol. 2025 Sep 19;15(9):e71825. doi: 10.1002/ece3.71825 (PMC12449034; doi:10.1002/ece3.71825)

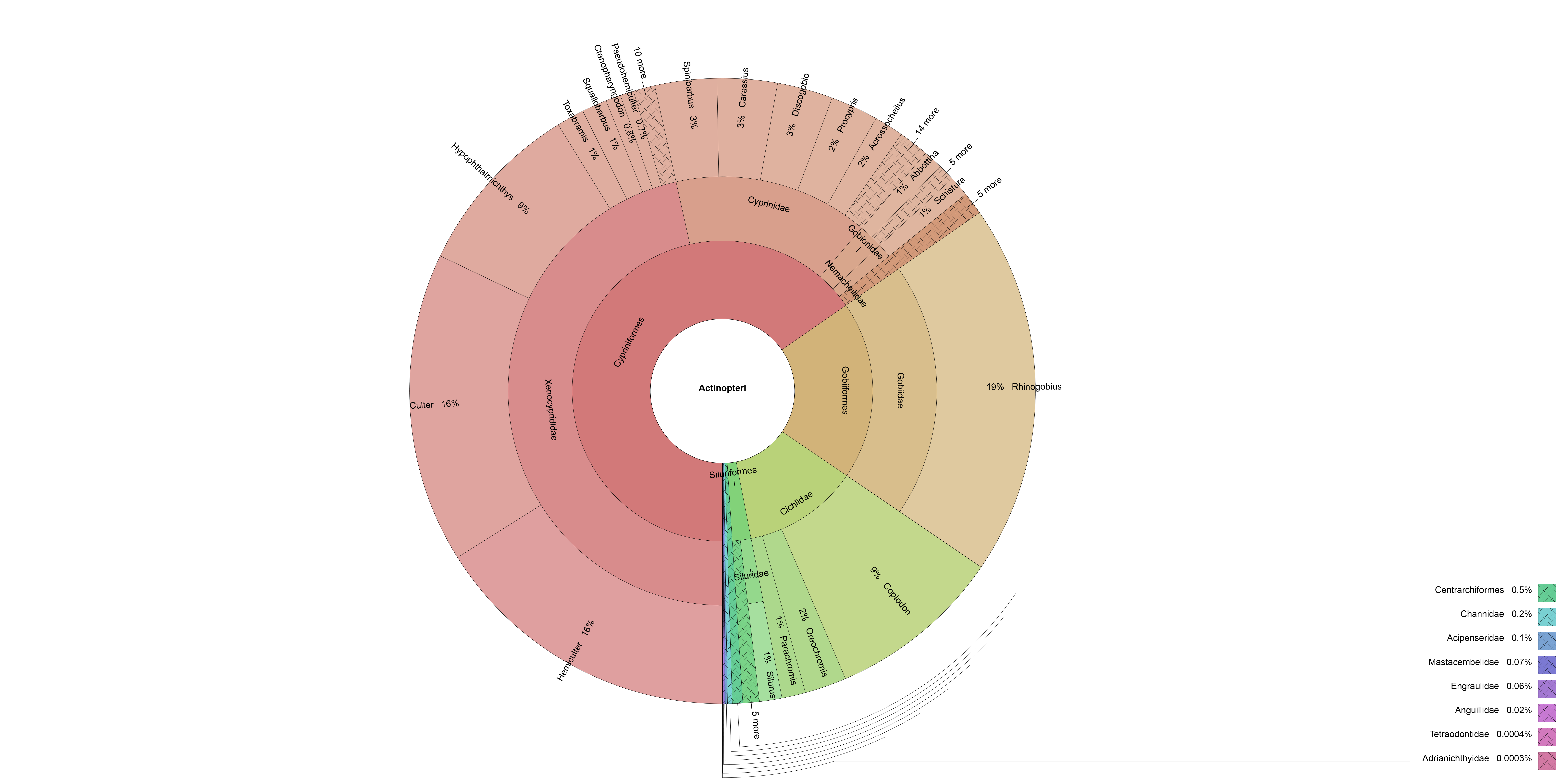

Supplement: Supplementary file 1 — Figure S1: ece371825‐sup‐0001‐FigureS1.tif. [file ECE3-15-e71825-s001.tif]

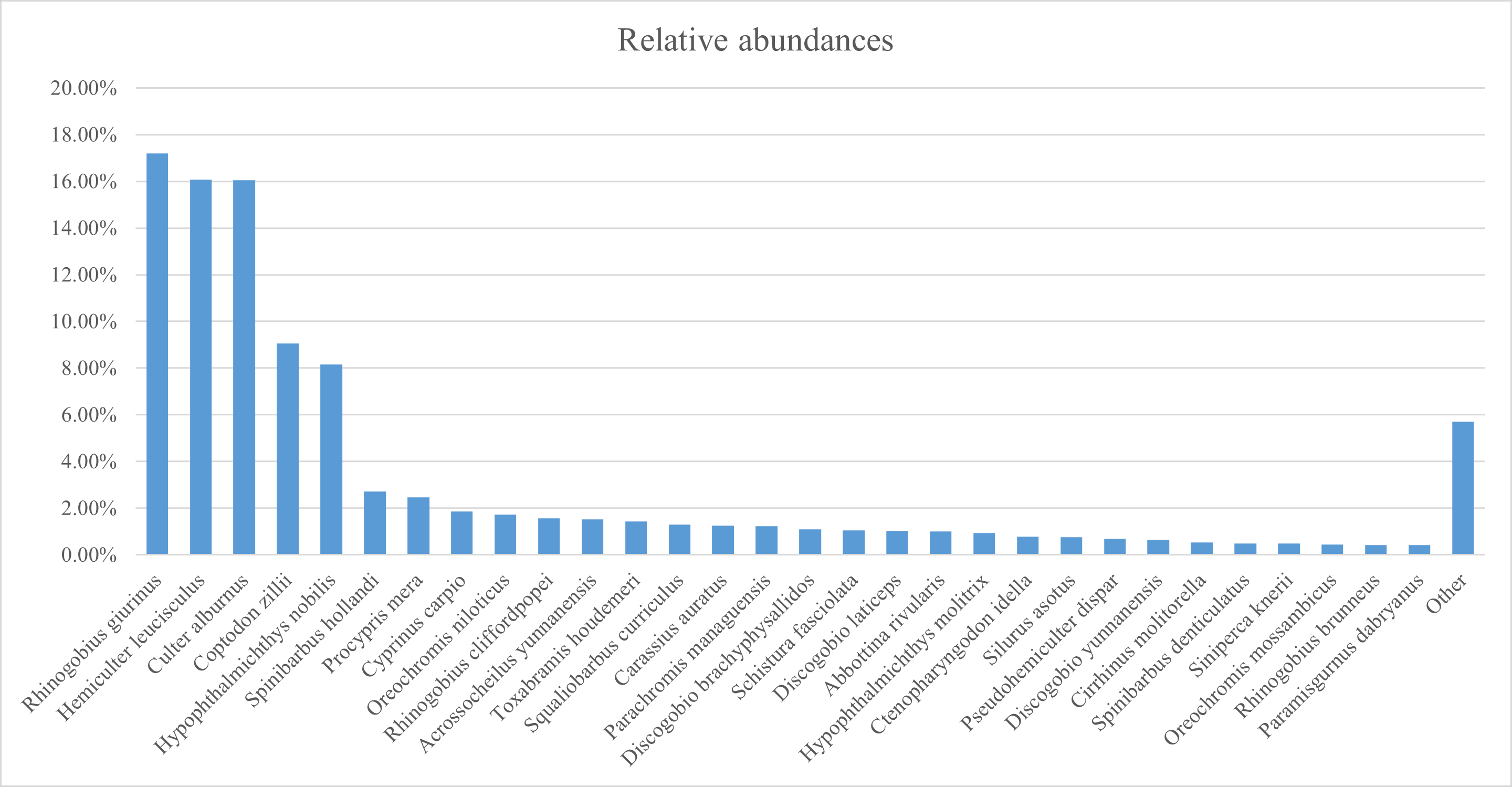

Supplement: Supplementary file 2 — Figure S2: ece371825‐sup‐0002‐FigureS2.tif. [file ECE3-15-e71825-s002.tif]

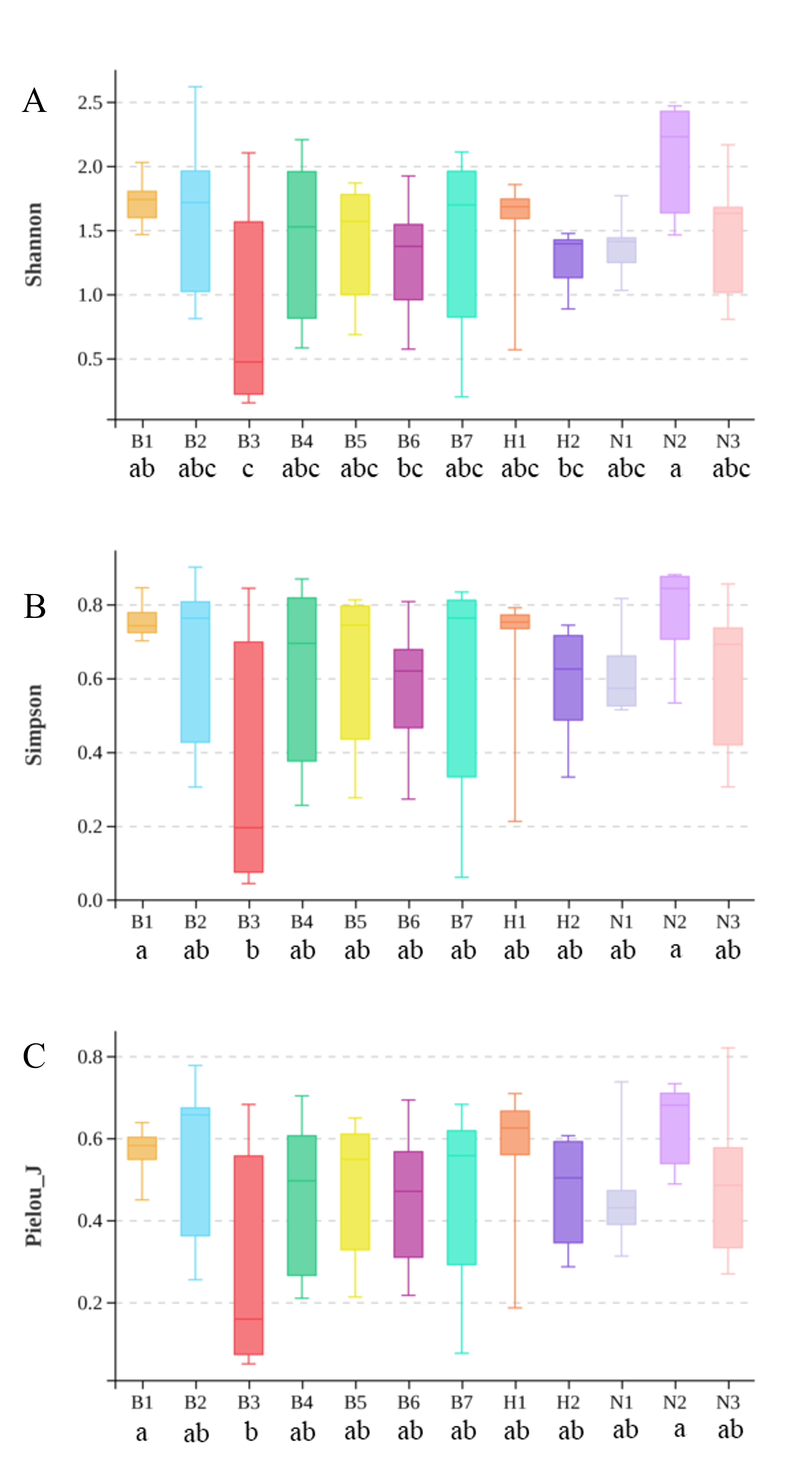

Supplement: Supplementary file 3 — Figure S3: ece371825‐sup‐0003‐FigureS3.tif. [file ECE3-15-e71825-s004.tif]
