# Supplementary material for: Environmental DNA Reveals the Fish Community Structure Exhibited Instability and Trend of Miniaturization in the Xijiang River Basin of the Guizhou
Source: Ecol Evol. 2025 Sep 19;15(9):e71825. doi: 10.1002/ece3.71825 (PMC12449034; doi:10.1002/ece3.71825)
Supplement: Supplementary file 4 — Table S1: ece371825‐sup‐0004‐TableS1.docx. [file ECE3-15-e71825-s003.docx]

**Table S1.** SIMPER (Similarity Percentages) Analysis results.

| taxonomy | average | sd | ratio | cumsum | contribution |
| --- | --- | --- | --- | --- | --- |
| *Rhinogobius giurinus* | 0.179267 | 0.163756 | 1.094722 | 0.195969 | 0.195969 |
| *Hemiculter leucisculus* | 0.106108 | 0.122068 | 0.869255 | 0.311964 | 0.115994 |
| *Culter alburnus* | 0.098222 | 0.227372 | 0.431989 | 0.419337 | 0.107374 |
| *Coptodon zillii* | 0.097273 | 0.085655 | 1.135634 | 0.525673 | 0.106336 |
| *Hypophthalmichthys nobilis* | 0.072197 | 0.148412 | 0.486463 | 0.604596 | 0.078923 |
